# Ancient role of *ten-m*/*odz* in segmentation and the transition from sequential to syncytial segmentation

**DOI:** 10.1186/s41065-017-0029-1

**Published:** 2017-04-27

**Authors:** Axel Hunding, Stefan Baumgartner

**Affiliations:** 1 Biophysical Chemistry, Department of Chemistry S01, H. C. 0rsted Institute, University of Copenhagen, Universitetsparken 5, DK-2100 Copenhagen, Denmark; 2 Department of Experimental Medical Sciences, Lund University, BMC D10, 22184 Lund, Sweden

**Keywords:** Segmentation, Cambrian explosion, Chemical oscillations, Ten-m, Ftz

## Abstract

**Background:**

Until recently, mechanisms of segmentation established for *Drosophila* served as a paradigm for arthropod segmentation. However, with the discovery of gene expression waves in vertebrate segmentation, another paradigm based on oscillations linked to axial growth was established. The *Notch* pathway and *hairy* delay oscillator are basic components of this mechanism, as is the *wnt* pathway. With the establishment of oscillations during segmentation of the beetle *Tribolium*, a common segmentation mechanism may have been present in the last common ancestor of vertebrates and arthropods. However, the *Notch* pathway is not involved in segmentation of the initial *Drosophila* embryo. In arthropods, the *engrailed*, *wingless* pair has a much more conserved function in segmentation than most of the hierarchy established for *Drosophila.*

**Results:**

Here, we work backwards from this conserved pair by discussing possible mechanisms which could have taken over the role of the *Notch* pathway. We propose a pivotal role for the large transmembrane protein Ten-m/Odz. Ten-m/Odz may have had an ancient role in cell-cell communication, parallel to the *Notch* and *wnt* pathways. The Ten-m protein binds to the membrane with properties which resemble other membrane-based biochemical oscillators.

**Conclusion:**

We propose that such a simple transition could have formed the initial scaffold, on top of which the hierarchy, observed in the syncytium of dipterans, could have evolved.

## Background

The idea that segmentation arose well before the Cambrian explosion, even before the last common ancestor of vertebrates and arthropods, has recently been under discussion [1–6]. For more than a decade, in attempts to understand sequential segmentation in other arthropods, the paradigm for *Drosophila* segmentation was used to explain the mechanisms of sequential segmentation. In parallel, other mechanisms proposed for vertebrates emerged, representing an alternative paradigm and being probably even closer related to sequential segmentation [3, 7–9].

At nuclear cycle 10 to 14, the *Drosophila* embryo is a syncytium with nuclei dividing in a layer close to the outer membrane. Since no cell walls have formed yet, protein gradients can arise through diffusion or active transport. Indeed, a hierarchy of such gradients gradually pattern the embryo [1, 2, 4, 10–12]. Maternal gradients from both ends of the embryo determine the locations of the proteins of the next level of the hierarchy, the gap genes. These arise in broad bands, and maternal and gap proteins in turn define region specific cues. At the next level of the hierarchy, the pair-rule genes are controlled by a combination of the gap genes, and the result is the emergence of the first repetitive pattern in the embryo, the seven striped pair-rule patterns of genes such as *hairy (h), even-skipped (eve)* and *runt (run)*. The pair-rule genes define the final level of the hierarchy, that of the segment-polarity genes such as *wingless* (*wg*) and (*engrailed*) (*en*), which emerge as 14 stripes, while cellularization is in progress. During this process, a membrane moves from the apical to the basal side of the nuclei and finally encases them.

**Fig. 1:**
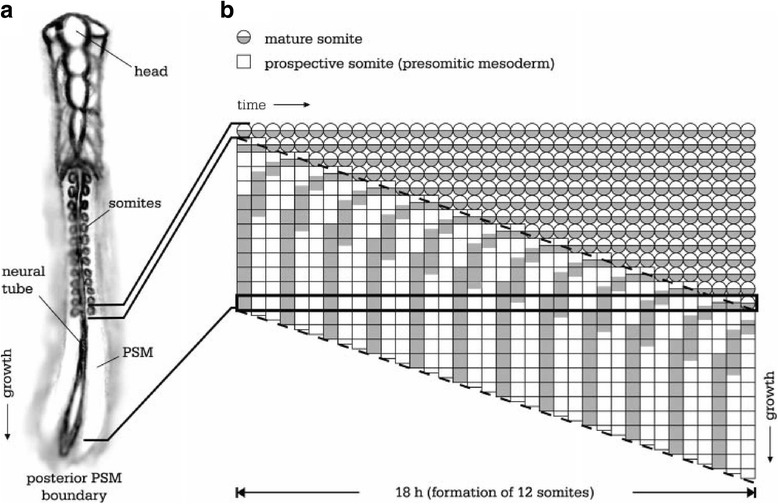
Somite formation in the oscillator-growth scenario. **a** a vertical embryo is depicted, with anterior head above and posterior tail region below, where the growth zone is located. The presomitic mesoderm (PSM) is the region from the last already-formed somite to the growth zone at the posterior PSM boundary. The time evolution of newly formed cells at the growth zone is depicted in (**b**) as the horizontal black bar. Initially, a gene expression, depicted as a grey time box, oscillates in time (with a period of three time-boxes, left part of *black* bar). Eventually (right part of the *horizontal* bar), the period increases to more than six time boxes, (and even more, see Fig. 5a) and the oscillation thus comes almost to a halt. A bistable system freezes this gene expression in the (almost) stopped phase, somite boundaries start to form and a new mature somite is created, depicted as a circle. Thus somites are added in an anterior to posterior direction, with control from the posterior part of the embryo. Reproduced from [58]

In vertebrates, a completely different mode operates during segmentation. Periodically-arising gene-expression waves, first established in chicken embryos [7], arise from the elongating posterior end of the embryo and run towards the anterior where they gradually stop, thereby adding a segment per period. Subsequently, this was interpreted as an oscillation under control of the chicken Hairy protein. This protein binds to its own promoter and inhibits its own activity, but due to a delay between formation of the corresponding mRNA and the protein, a biochemical oscillator emerges [13]. At the posterior end, cells are converted into the presomitic mesoderm (PSM) during the oscillation mode (Fig. 1). As the embryo elongates axially, new oscillating cells emerge in the PSM with varying phases. Eventually, the period of oscillation of an individual cell becomes larger, until the oscillation eventually stops and segment borders form. This process repeats itself when the next group of cells come to a halt during their oscillation, and thus segments form sequentially (from anterior towards posterior).

How the periodic spatial pattern generated by gene-expression waves are translated into actual somites is still a matter of discussion. The high and low protein concentrations must interfere with a bistable mechanism which is capable of storing these phases. The same problem arises in arthropod segmentation. In *Drosophila*, the *wg, en* and (*hedgehog*) *hh* module maintains the spatial pattern once formed [14].

The essential traits of the vertebrate segmentation paradigm is an oscillator coupled to axial growth, slowing of the oscillation, and stabilization by a bistable switch. Although often referred to as a ‘clock-and-wave front’ mechanism, the original CW-model [15] was assigned a role for the control of segmentation from the anterior part of the embryo. However, a modification proposed by Newman with a clock running in the posterior growth zone, with the period frozen when cells entered the PSM [16], is closer to our current thinking. Indeed, this mechanism may be relatively easy to achieve initially during early evolution. This mechanism would yield almost instantly-formed metameric units emanating from the posterior end. However, if there was an advantage of showing the freezing of the oscillation phase postponed, a control system could gradually evolve to achieve this feature.

The *Notch* pathway was shown to be involved in spider segmentation (a basal arthropod) [17]. This report opened a new door to arthropod segmentation. It was speculated that this mechanism of segmentation may have been ancestral, predating the last common ancestor of vertebrates and arthropods. This argument was further strengthened by the establishment of an important role for the *Notch* pathway in cockroach and cricket segmentation [18, 19]. Somewhat later, a role for *Notch* at the root of insects for segmentation of the crustacean *Artemia franciscana* was reported [20]. However, the reports on the significance of the *Notch* pathway in arthropod segmentation were recently challenged again [21–23].

Recently, further progress in understanding oscillator mechanisms in arthropods was made by the demonstration of the existence of a segmentation clock in the *odd-skipped* (*odd*) and *eve* gens in the flour beetle *Tribolium* which supports the notion of a clock-based mechanism in both vertebrates and arthropods [24–26].

However, a common clock based on the *Notch* pathway must have been lost in the evolution of insects, as the *Notch* pathway is not involved in early embryonic segmentation in *Drosophila*, although it is involved later during development in segmentation of the appendages. Likewise, *Notch* signaling does not regulate early segmentation in the honeybee, a basal holometabolous insect [27].

The role of the *Notch* pathway was originally described to have a share in the oscillating mechanism, but alternatively, it may function to signal locally between cells, thus synchronizing these [28]. Differences exist between zebrafish, chick and mouse somitogenesis, but the essential feature is an oscillator system coupled to axial growth. An interplay between the *Notch*, *wnt* and *FGF* pathways was established [29, 30], however, recent results indicated that a component was missing in the oscillating mechanism [8, 31, 32].

The vertebrate segmentation paradigm introduces another framework for evaluating experimental data of basal sequential insect segmentation which otherwise were interpreted to comply one way or another with the *Drosophila* paradigm. Notably, some of the gap genes may be involved in axial elongation, rather than defining zones of segments. Truncation may occur [33, 34], but truncation may also be due to misregulation of pair-rule genes. A reinterpretation of the role of gap genes, however, does not give any clear answers [4]. The role of the primary pair-rule genes is quite variable, sometimes revealing a pair- rule function, sometimes a function like segment-polarity genes, sometimes neither of these [35–37]. The *wg, en and hh* module has a much more conserved function in defining segment borders. The observation that many pair-rule genes also harbor regulatory elements for segmental expression led to the (meanwhile) revised conclusion that the most widely-conserved role of the pair-rule genes may be at the single-segment level, and not at the double-segment level [2, 35, 38].

In this context, it is noteworthy to mention that not all insects follow the same mode of segmentation. In *Drosophila*, the patterns of expression of segmentation genes are established simultaneously in all segments by a complex set of interactions between transcriptional factors that diffuse in a syncytium occupying the whole embryo [39, 40]. Such mechanisms cannot act in short germ-band insects such as the grasshopper *Schistocerca* where the blastoderm initially comprises only one segment, and the remaining segments are sequentially produced from a posterior proliferative zone. The most widespread mode of segmentation among insects is found in the intermediate germ-band organisms such as *Tribolium*, where a species-specific number of segments forms synchronously from an anteriorly restricted blastoderm, whereas the other tissues form sequentially from a posterior proliferative zone.

The nature of the oscillator which could play the role as a possible replacement for the *Notch* pathway is still unsolved. We wish to propose an alternative mechanism. To this end, we worked backwards from the *wg, en, hh* module and searched for genes functionally close to this module which could have a role as a presumptive oscillator and at the same time showing a function in cell-cell communication. We wish to argue that the *ten-m* gene [41] (also called *odz* [42]) has many properties which may place this gene in a central role for ancient segmentation, and possibly in arthropod segmentation, as well. *ten-m* is involved in segmentation in both vertebrates and insects [43]. This gene encodes a large type II transmembrane protein which is bound to the cell membrane. In *Drosophila*, it is located on the inwards-growing membrane which intercalates the nuclei in the syncytium. A long extracellular part of the protein is involved in homodimerization, and the dynamics of this process has properties which may create a biochemical oscillator. Homophilic interactions of Ten-m on the membrane eventually induce cleavage of Ten-m on the intracellular small part, which translocates to the nucleus. A link between *ten-m* and *zic,* the vertebrate homolog of *odd-paired (opa)*, was discussed [44]. In *Drosophila*, *ten-m* is not transcribed in pair-rule stripes, rather the mRNA is expressed fairly homogeneous, but after translation the protein forms seven stripes at early gastrulation ([41], Fig. 6). The mechanism for this observation is so far unexplained, but we propose that the internal dynamics of the protein-membrane interactions will set up a spontaneous pattern-forming mechanism, well known from similar biochemical control systems. Interestingly, despite the fact that it shows a striped expression, *ten-m* mutants show a normal cuticle, however, most *ten-m* mutants die at the first larval stage [45].

The role of the equidistantly-striped Ten-m protein in *Drosophila* is presently unknown. However, the presence of the stripes during late cycle 14 and early gastrulation is early enough to provide an equidistantly striped pattern, which may complement the broad subdivision provided by gap gene cues.

We wish to propose that the oscillatory properties of Ten-m may play a seminal role in arthropod segmentation. Finally, we propose a mechanism by which Ten-m would play an important role in cell-cell communication and in axial sequential segmentation in lower arthropods, by a simple parameter change in the control system to an all-at-once pattern forming mechanism. We propose that such a simple transition could have formed the initial scaffold, on top of which the hierarchy, observed in the syncytium of dipterans, could have evolved.

## Results

### Membrane oscillator and bistability

The vertebrate oscillator, coupled to axial growth, was proposed to arise from self-inhibition of the Hairy protein which binds to its own promoter [13]. Thus, synthesis of *hairy* mRNA is inhibited by Hairy protein, which is itself translated from the mRNA. Such a two-component inhibitory feedback system is only an oscillator, if a delay is invoked in the mechanism.

Our proposed Ten-m oscillator does not have its origin in a delayed translation, but arises from cooperative membrane binding. After transcription and translation, the Ten-m protein in the cytoplasm binds to the cell membrane. Subsequently, the bound form in the membrane reacts to create homodimers. If the uptake to the membrane is enhanced in regions with homodimers, cooperative binding of the cytoplasmic form will take place. We presume cooperative binding in our model, presented in the section “Models” below.

**Fig. 2:**
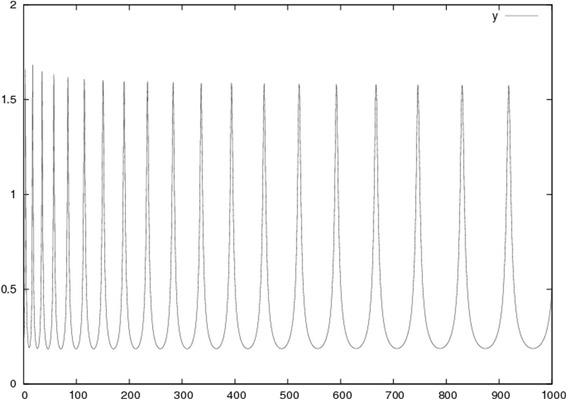
The Ten-m oscillator. In the mathematical model, Eqs. (3,4), *k*_*2*_ is varied from 0.75 to l/100th of this, simulating control from FGF8. The Hill coefficient $\gamma$ is 2, and the other Hill coefficient *m* is 1.25, initially, approaching $\gamma$ when *k*_*2*_ goes down. *k*_*3*_ is taken initially to 0.49, close to *k*_*3c*_ (Eq. 15), and when *k*_*2*_ varies by two orders of magnitude, *k*_*3*_ varies with a modest factor 3.5. The result is a relatively rapid oscillation at first, with *T*_*p*_ = 10.5, but gradually, the period grows and reaches values of ~ 10 times larger than in the beginning. In experimentally observed gene expression waves, the period grows with a factor of about 6, after which the oscillation is trapped by a bistable system

The homodimers are known to induce cleavage of the intracellular short part of the protein, perhaps through interaction with receptor tyrosine kinases. In vertebrates, it was found that the short part of the protein translocates to the nucleus where it acts as a transcriptional repressor of *zic,* a vertebrate homologue of *opa* [44]. Conversely, ubiquitous Zic causes rapid degradation in the cytoplasm of the short intra Ten-m. In the manuscript, we refer to *opa/zic* where appropriate for the convenience of the reader. Through modeling of the described cooperative binding of Ten-m to the membrane accompanied with subsequent degradation, it is observed that a biochemical oscillator can emerge (Fig. 2). The model is described in detail in the section “Models”, where parameters are discussed.

In vertebrates, it was shown that a protein gradient of FGF8 emerges due to *fgf8* transcription in the posterior growth zone and subsequent mRNA degradation within cells in the PSM [46, 47]. This gradient causes the oscillation to cycle slower. A similar gradient of Wnt is also present. However, none of the three signaling *Notch*, *wnt* or *FGF* pathways, when activated in the PSM individually, appear to act as the global pacemaker [31, 32]. Links between the *Notch*, *wnt* and *FGF8* pathways were suggested and modeled [8, 29]. FGF8 is also coupled to the role of *ten-m* in vertebrate limb formation and possibly in segmentation as well [43, 48]. However, the mechanism of how to slow down the period of the oscillation is still unsolved [43]. Recently, however, it was shown that intercellular coupling through the *Delta-Notch* pathway has some role in the regulation of the period [49].

The region where the phase of the slowed down oscillator becomes fixed (the so-called determination front in vertebrates) is coupled to opposing retinoic acid and FGF gradients, but the link to a bistable system is not well established in vertebrates (see [50] for a model). Nonlinear bistable biochemical switches generally have the property that their dynamics allow two mutually-exclusive stable stationary states, one in which a component A is high and another component B is low, and another with high B and low A. The dynamics may be such that the module has only one (possibly symmetrical) state, if a control parameter is below a critical threshold, but enters the bistable regime when the control parameter exceeds the threshold. Which of the two states is then selected (high A, low B or vice versa) depends on small fluctuations in the dynamics. If the system is biased beforehand towards an excess of, say A, then entering the bistable regime with the control parameter exceeding the threshold will select the high A/low B state. Thus, if the gene expression oscillator is coupled to such a bistable system, and the slow-down of the oscillation occurs in cells which have entered the bistable regime, then the phase of the oscillator may bias the bistable system to select (and maintain) this phase for subsequent times.

### Period doubling

**Fig. 3:**
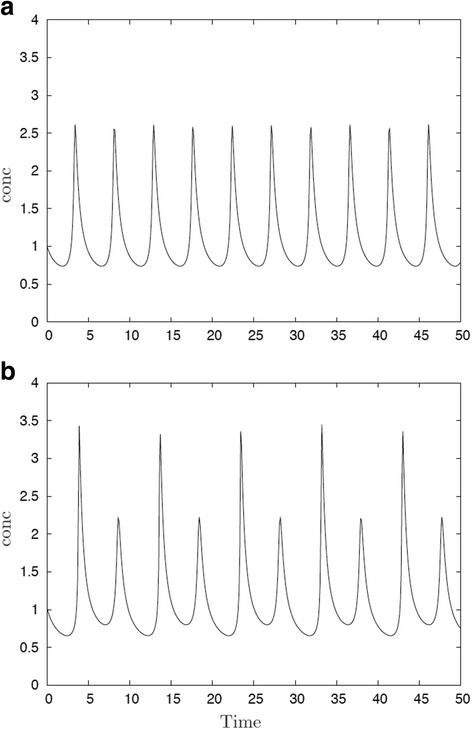
Period 2 oscillation. **a** a normal oscillator is seen. Concentration oscillates with a period *T*_*a*_ of about 4.5 time units. **b** the system has undergone period-doubling. Former high peaks with period *T*_*a*_ are replaced by high peaks only at every second peak, with a lower intercalating peak

It is a commonly observed feature of autonomous nonlinear oscillators that a phenomenon known as period-doubling occurs [51]. The phenomenon may arise in several ways, e.g. if more than one oscillator is present and coupled, or a delay or a feedback in the mechanism is introduced [52]. Whereas the original system may have regular peaks entering with period *T*_*0*_ *,* the coupled system may modify the oscillation such that only every other peak emerges with high amplitude, whereas in between, the former high peaks are somewhat reduced, (Fig. 3). The system is still periodic, but now with a period closer to 2 *T*_*0*_ *.* Such a system would still give rise to a full set of segments laid down with period *T*_*0*_ *,* but the higher period-2 peaks should be capable of triggering the bistable stabilizing system well before the smaller peaks in between. In the axially-growing embryo, one would then observe gene activation in a double segment periodicity, and somewhat later complemented with intercalating gene activation in between the first wave of stripes.

Such a control system could even be used as a scaffold for separating the promoter control for the first wave of stripes with double-segment periodicity, and the second wave of intercalary stripes. Note also that genes involved in such an oscillator system may easily change roles (by small parameter changes in the control) from a ‘segment polarity gene’ (oscillator with period *T*_*0*_) to a ‘pair-rule gene’ (period doubled oscillator with period 2 *T*_*0*_), and even to a gene not involved in stripe formation (parameter change of the control system to a non-oscillatory regime). Thus, the apparent variability of the function of primary pair-rule genes such as *eve* in lower arthropods, with pair-rule expression only (but not segmental expression), segmental only, both kind of expressions or neither of them [35–37] can be controlled by minor changes in the oscillator control system.

The possible relevance of the phenomenon of period-doubling in oscillating systems, as outlined above, has not been implemented in an explicit model by us, since presumptive couplings of the ten-m oscillator to other autonomous oscillators (like those in the Notch, Wnt and FGF pathways) are so far a matter for future research.

In the next section, we discuss the quantitative aspects of the proposed oscillator, based on cooperative Ten-m binding to the membrane.

### Models

The dynamics of Ten-m is described in analogy with an earlier model by [53], which is further akin to a model by [54] on protein binding to the cell membrane, in the context of prokaryotic cell division. The main feature of this model has been experimentally verified with in vitro experiments and further modeling [55].

We will use linear stability theory to show that the model comprises an autonomous oscillator, and find parameters relevant to the onset of oscillations. Generally, such an analysis may be performed by a linearization of the proposed rate-laws. From the elements *a*, *b, c, d* of the Jacobian (see below), one obtains the characteristic equation

1 $$ {\lambda}^2+ T r\lambda + det=0 $$

with *Tr = a + d* and det = *ad* — *bc.* The eigenvalues $\lambda$ found determine the fate of small perturbations from the stationary state: if the real part of $\lambda$ is found negative, the perturbations relax or spiral into the stationary state, and the system is locally stable. If the real part is positive, the perturbations are amplified, and the stationary state is locally unstable. The conditions *Tr* < 0 and det > 0 together guaranty local stability, according to the general theory. Onset of oscillations may occur if the condition *Tr* < 0 is violated. At the transition (where *Tr =* 0), Eq. (1) becomes

2 $$ {\lambda}^2+ det=0 $$

The imaginary part *Im* of $\lambda$ is thus √det which allows solutions of the perturbations of the form cos*(Imt)*. From this the period, *T*_*p*_ of these oscillations can be determined since 2$\pi$= *ImT*_*p*_ *=√ detT*_*p*_.

Thus, in our model we use

3 $$ x'={k}_1\hbox{--} 2{k}_2 x{y}^{\gamma} $$

4 $$ y'={k}_2 x{y}^{\gamma}\hbox{--} {k}_3 y $$

Here, *x* is the cytoplasmic concentration of the Ten-m protein, and *y* is its membrane-bound dimer. We assume a constant rate *k*_*1*_ of synthesis of *x,* and a Hill-type uptake-rate proportional to *y* ^*γ*^/(*L* + *y* ^*γ*^), in analogy with the above models. For simplicity, we only keep the nominator in order to find analytical expressions for parameter values at the onset of oscillation or pattern formation. A new feature is the introduction of Hill-type degradation of *y* as well, so we rewrite *k*_3_ *y* to *k*_3_ *y* ^*m*^.

The elements of the Jacobian evaluates to

5 $$ a=\delta \left({x}^{\prime}\right)/\delta (x)=-2{k}_2{y}^{\gamma} $$

6 $$ b=\delta \left({x}^{\prime}\right)/\delta (y)=-2{k}_2 x\gamma {y}^{\left(\gamma -1\right)} $$

7 $$ c=\delta \left({y}^{\prime}\right)/\delta (x)={k}_2{y}^{\gamma} $$

8 $$ d=\delta \left({y}^{\prime}\right)/\delta (y)={k}_2\gamma x{y}^{\left(\gamma -1\right)}-{k}_2 m{y}^{\left( m-1\right)} $$

The determinand of the Jacobian det = (*ad-bc*) evaluated at the stationary state yields

9 $$ det=\left(-2{k}_2{y}^{\gamma}\right)\left({k}_2\gamma x{y}^{\left(\gamma -1\right)}-{k}_3 m{y}^{\left( m-1\right)}\right)-\left(-2{k}_2\gamma x{y}^{\left(\gamma -1\right)}{k}_2{y}^{\gamma}\right)=2{k}_2{k}_3 m{\left(\frac{\left({k}_1\right)}{\left(2{k}_3\right)}\right)}^{\left(\frac{\left(\gamma -1\right)}{m}\right)}\frac{k_1}{\left(2{k}_3\right)}={k}_1{k}_2 m{\left(\frac{k_1}{\left(2{k}_3\right)}\right)}^{\left(\left(\gamma -1\right)/ m\right)} $$

where

10 $$ {y}^m=\frac{k_1}{\left(2{k}_3\right)} $$

and the two terms with *xy* ^(*γ*-1)^ cancel each other. The determinand is thus always positive.

The system will be an oscillator provided

11 $$ T r= a+ d=-2{k}_2{y}^{\gamma}+{k}_2 x\gamma {y}^{\left(\gamma -1\right)}-{k}_3 m{y}^{\left( m-1\right)}>0 $$

Multiplying with *y*

12 $$ -2{k}_2{y}^{\left(\gamma +1\right)}+{k}_2 x\gamma {y}^{\gamma}-{k}_3 m{y}^m>0 $$

and thus

13 $$ {k}_2\gamma \frac{k_1}{\left(2{k}_2\right)}>2{k}_2{\left(\frac{k_1}{\left(2{k}_3\right)}\right)}^{\left(\left(\gamma +1\right)/ m\right)}+ m\left({k}_1/2\right)>0 $$

which yields

14 $$ \gamma - m>4\frac{k_2}{k_1}{\left(\frac{k_1}{\left(2{k}_3\right)}\right)}^{\left(\left(\gamma +1\right)/ m\right)} $$

and thus a positive value for the difference *γ* -*m* in cooperativity.

This may be rearranged to

15 $$ {k}_3>{k}_{3 c}=\frac{k_1}{2}{\left(4\frac{k_2}{\left({k}_1\left(\gamma - m\right)\right)}\right)}^{\left( m/\left(\gamma +1\right)\right)} $$

The period *T*_*p*_ of this oscillation may be estimated for *k*_3_ > *k*_3*c*_ (i.e. close to the Hopf bifurcation point) from the value of the determinand as

16 $$ {T}_p\simeq 2\frac{\pi}{\sqrt{(det)}}=\frac{\left(2\pi \right)}{{\left( m{k}_1{k}_2\right)}^{\left(1/2\right)}}{\left(\frac{\left(4{k}_2\right)}{\left({k}_1\left(\gamma - m\right)\right)}\right)}^{\left(\frac{\left(\gamma -1\right)}{\left(2\left(\gamma +1\right)\right)}\right)} $$

One desired property of oscillators related to sequential segmentation is that the control of the period (e.g. by a gradient in FGF8, see main text) should be feasible without changing too many parameters drastically simultaneously. In principle, FGF8 could decrease all three reaction constants *k*_*1*_, *k*_*2*_ and *k*_*3*_ with the same magnitude. In this case, Eqs. (3) and (4) simply rescale to a new time. In reality, this seems highly implausible. To increase *T*_*p*_ with only a major change in one parameter, but modest changes in the rest is more difficult to achieve. For example, only increasing *k*_*3*_ to a value far from the bifurcation point may lead to a long period, but simultaneously to a high- amplitude, highly nonlinear saw-tooth type oscillation.

However, from the expression Eq. (13), the power ($\gamma$ — l)/(2($\gamma$ + 1)) evaluates to 1/6 for a realistic Hill constant $\gamma$ = 2. This means that the argument $\eta$ *= 4 k*_*2*_ */k*_*1*_ ($\gamma$ — *m))* raised to this power may change relatively modestly. Indeed, due to the front factor *2π*/(*mk*_*1*_ *k*_*2*_)^*1*/*2*^, the period *T*_*p*_ becomes largely inversely proportional to the square root of *k*_*2*_ and thus decreasing *k*_*2*_ a factor of 25 from, say, 0.5 to 0.02 would result in a five-fold increase in the period, provided *η* ^1/6^ does not change much. If the cooperatively *m* approaches $\gamma$ in this transition, *η* ^1/6^ would change even less.

It is thus possible to increase the period with a constant *k*_*1*_, a substantial (FGF8-induced) change in *k*_*2*_ and a modest increase in the degradation cooperativity *m* from, say, 1.25 to a value closer to $\gamma$ = 2 such that $\eta$ does not change a lot. Hereby, the degradation rate constant *k*_*3*_ may also remain almost constant. A simulation of this increase in oscillation period by control of *k*_*2*_ is given in Fig. 2.

Our assumptions of the changing constants may appear odd at first. But starting from a Newman type mechanism with instant freezing of the phase of the oscillator, when cells enter the PSM (see above), it is possible to have gradual improvements during evolution towards a mechanism with oscillator slow-down. There is probably an advantage, although hitherto not discussed in the literature, to have a gradual slow-down of the oscillator along the PSM, instead of instant freezing. In our model, the sigmoid form of degradation could evolve from a simple first-order kinetics, but with a Hill constant *m* always lower than $\gamma$ (otherwise oscillations stop, as shown). [56] describes an example of such a cooperative degradation. This would lengthen the period, but to achieve an increase of the period with as much as a factor 6 (as in Fig. 2), *k*_*3*_ has to be changed modestly as well, if large amplitudes are to be avoided. However, when *k*_*2*_ is decreased, a decrease in $\gamma$ — *m* (i.e. *m* approaching $\gamma$) to about the value of the diminished *k*_*2*_ would keep *k*_*3*_ virtually constant. Such a scenario is possible during evolution, as it is a gradual process retaining the oscillatory dynamics throughout.

As eluted above, metameric structures are possible in an oscillator-growth scenario based on Ten-m. The transition from such a sequential stripe-forming mechanism to an all-or-none Turing mechanism may first require a syncytium. The advantage of a syncytium has not been much discussed in the literature, except for the observation that pattern formation may be much faster, if diffusion in a common cytoplasm replaces cell-cell communication. A role for a syncytium in an oscillator-growth scenario may also be ascribed to the need for local synchronization of the oscillators.

The transition from sequential to all-or-none stripe formation may be discussed in terms of the Turing wave length of the syncytial system. Here, *x* is the rapidly diffusing cytoplasmic component, while *y* is the membrane-bound slowly diffusing species. The characteristic wave length $\lambda$_*T*_ of such a system is *λ*_*T*_ = *2π*/*κ*_*T*_ with *n*_*T*_ [57] and is given by

17 $$ {\kappa}_T^2=\frac{\left( a{D}_y+ d{D}_x\right)}{\left(2{D}_x{D}_y\right)} $$

with *D*_*x*_ and *D*_*y*_ the diffusion coefficients of *x* and *y* .

**Fig. 4:**
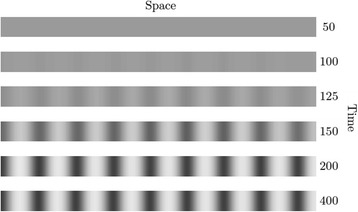
Turing stripe formation in the Ten-m system. If *k*_*3*_ is below 0.50, homogeneous oscillations do not form, but if *D*_*x*_ *>> D*_*y*_ the system may be prone to spontaneous spatial pattern formation. Other parameters: $\gamma$ = 2.4, *m =* 1.6, *k*_*2*_ = 0.2, *k*_*3*_ *=* 0.475, *D*_*x*_ *=* 1.45 and *D*_*y*_ = 0.09. Once *k*_*3*_ *>* 0.53, homogeneous oscillations emerge, despite the large spread in diffusion constants (not shown here)

Figure 4 displays the emergence of a Turing stripe pattern in such a syncytial system. Since the Jacobian elements *a* and *d* derived above are proportional to *k*_*2*_ and $\gamma$ — *m*, respectively, and since both are diminishing in concert in the proposed model, *a* or *d* will not outgrow each other in Eq. (14). As *D*_*x*_ *>> D*_*y*_, one obtains the standard result that $\kappa$_*T*_ is approximately given as

18 $$ \kappa {2}_T\simeq \frac{d}{\left(2{D}_y\right)}=\frac{k_1}{\left(4{D}_y\right)}\left(\gamma - m\right){\left(\frac{\left(2{k}_3\right)}{k_1}\right)}^{\left(1/ m\right)} $$

**Fig. 5:**
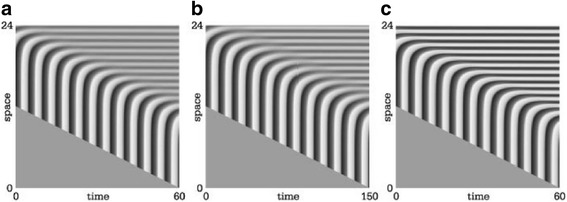
Transition from oscillator-growth to reaction-diffusion pattern formation. **a** standard oscillation-growth scenario, as seen in Fig. 1. **b** embryo parameters are changed and the oscillator-growth system is on the border of a transition to a reaction-diffusion based pattern-forming system in the mature (upper, right) part. **c** parameter transition is complete. The mature part of the embryo now forms a repetitive pattern by a R-D mechanism, but forcing from the oscillator in the posterior growth zone yields the *same* pattern as before the transition. Thus, the “mutant” embryo may survive this transition to another pattern-forming mechanism, which may then be exploited by evolution as a basis for a simultaneous all-or-none stripe mechanism. Reproduced from [58]

As usual, *λ*_*T*_ = 2*π*/*κ*_*T*_ becomes proportional to the square root of *D*_*y*_, and so depends on the slowly-diffusing, membrane-bound protein component. This diffusion may be much slower than free protein diffusion in the cytoplasm. However, in the related model (with MinE protein binding to membranes [55]), spatial wave lengths were found experimentally in the order of 50–70 *μm*. This is of the same order of magnitude as stripe separation in *Drosophila*, see also the discussion on Fig. 5.

For a specific parameter set, a spectrum of different $\kappa$’s is present. If Turing structures form from a (nearly) homogeneous state, the emerging pattern has a wave length close to *λ*_*T*_ = *2π*/*κ*_*T*_, see Eq. (18). However, if at the outset the homogeneous state is biased towards a certain wave length within this spectrum, this pattern may be the fastest growing mode. In Fig. 5, the transition from a time oscillator mechanism to a Turing mechanism by the change of a single parameter initially yields a spatial pattern with wave length determined by the oscillator-growth scenario. At the transition to the new pattern forming mechanism, this spatial pattern biases the Turing mechanism to grow with the same wave length, provided it is included in the Turing spectrum. One may note that the Turing wave length (which is roughly in the middle of the spectrum) yields $\lambda$_T_ $\alpha$ 1($\gamma$ — *m*)^1/2^. This change is of the same form as the period for the time period *T*_*p*_ in the oscillating-growth scenario and thus may be about an order of magnitude. During this change, the Turing system may be activated on the segment length induced by the posterior forcing oscillator outside the PSM (for a further discussion, see [58]).

### Experimental evidence of stripe formation

**Fig. 6:**
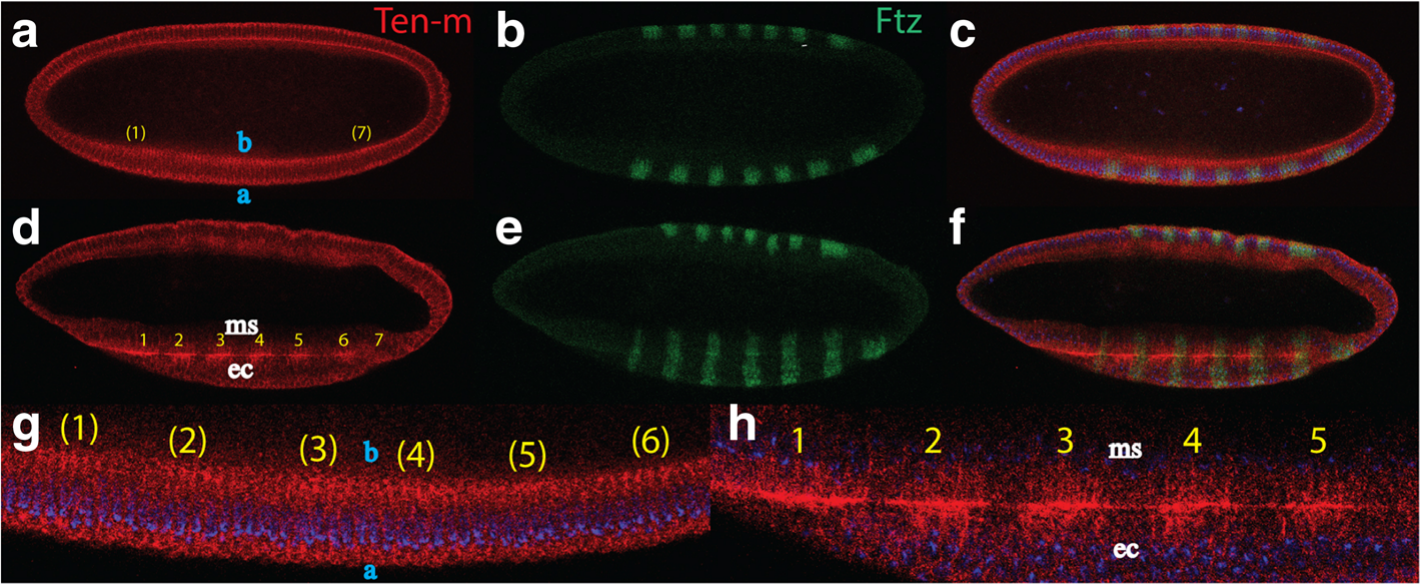
Formation of the Ten-m stripes at gastrulation. **a**-**c** confocal picture of a cellularized *Drosophila* embryo, anterior is to the left and dorsal side up. **a** Ten-m staining, the protein is detected at the basal (**b**) as well as apical (**a**) surface with stronger staining at the basal side where some presumptive stripes emerge, indicated by yellow colors, better seen in a magnification in (**g**). **b** identical embryo as in (**a**), stained for Ftz. The 7 stripes are already established. **c** merge of (**a**) and (**b**). **d**-**f** confocal picture of a *Drosophila* embryo at early gastrulation, anterior is to the left and dorsal side up. **d** 7 stripes with a width of about 4 cells and a gap of 4 cells have emerged which show strongest accumulation at the interphase between cells at the ectoderm (ec) and the mesoderm (ms), apart from being expressed at the other parts of the cell surface, best seen in a magnification of (**d**) in (**h**). **e** Identical embryos as in (**d**), Ftz stripes staining both the ectoderm. **f** merge of (**d**) and (**e**)

As discussed before, the *ten-m* RNA is expressed uniformly at the cellular blastoderm stage [41], but as soon as cellularization is finished and gastrulation commences, seven evenly-spaced stripes emerge. To monitor the formation of the stripes, we stained *Drosophila* embryos with anti-Ten-m antibodies and monitored the evolution of the stripes (Fig. 6). As an internal reference gene, we used the Fushi-tarazu (Ftz) protein. At the time when cellularization is completed, i.e. when each nucleus is encapsulated by a membrane, the first occurrence of a periodic Ten-m pattern was observed (Fig. 6a, g) where the protein was located at the basal as well at the apical surface of the blastoderm cells. Notably, the basal side only showed the emergence of the stripes (Fig. 6g), while the apical side showed uniform staining. In contrast to Ten-m, the stripe of the nuclear Ftz protein were already fully established at cellular blastoderm (Fig. 6b, c). About 30 min later, i.e. during gastrulation, where parts of the future mesoderm cells on the ventral side migrated to the interior of the embryos, Ten-m protein was accumulated in seven stripes at the basal side of the two cells layers, ectoderm and mesoderm (Fig. 6d, h) suggesting that homophilic adhesion of Ten-m between mesoderm and ectoderm cells occurred in a striped fashion. Since Ten-m was not found in stripes on the apical side, it follows that the formation of the stripes must be controlled at the cellular level, and only the basal part was involved in the proposed mechanism of periodic stripe formation. Of note is the fact that the *ten-m* RNA was still expressed uniformly at this stage [41]. In contrast to Ten-m, the Ftz protein was expressed in nuclei in the entity of the two cell layers (Fig. 6e, f), and did not show any distinct pattern between the ectoderm and the mesoderm.

## Discussion

### The protostome-deuterostome last common ancestor

The early evolution of multicellular animals after some 1500 Ma of successful unicellular eukaryotes yields a much debated animal tree of life [59–64]. The complexity of the last common ancestor to protostomes and deuterostomes is also a matter of much controversy [65, 66], as is the role of segmentation [67–69]. A segmented common ancestor implies either substantial gene loss, or at least loss of function, notably in unsegmented protostomes [70, 71]. The case for a common segmented ancestor has been reviewed in [6].

However, most biologists consider the high degree of similarity of many processes to be the result of independent recruitment of an ancient toolkit, rather than favoring a complex ancestor [72–74]. The principle of the segmentation clock may even be conserved across the animal and plant kingdoms, and thus comprises a universal design principle (rather than a common ancestor), as argued by [75].

Several cell-cell interaction genes are very ancient, like those of the *Notch, wnt, TGF* and *hedgehog* pathways [65]. The proposed Ten-m oscillator may have originated from proteins forming a cell-cell interaction system already in sponges. Indeed, a tenascin-like protein is found in Homoscleromorphs in the context of formation of basement membranes [76], which may constitute a key step in the evolution of true epithelia [60]. Precursors of the *ten-m* gene have been found in choanoflagellates [77]. Thus the *Notch, wnt, TGF* and *hedgehog* pathways together with *ten-m* family may have been ancient cell-cell interaction systems, and linking such pathways with receptor tyrosine kinase-based control of transcription factor function may have been an important innovation [59].

Cross-talk among cell-cell communication systems also occurs between the *Notch*, *wnt* and *zic* pathways [78] during neural development, which suggests that the Ten-m, Opa/Zic interaction could be a redundant communication system, functioning in parallel with Notch and/or Wnt. This opens the possibility for the key suggestion that the *ten-m, opa/zic* system may have replaced *Notch* as a cell-cell synchronization system early during evolution of arthropod segmentation.

To realize such transitions, another scenario for evolution of the syncytial stripes in *Drosophila* from a cellular system, based on an oscillator coupled to a bistable switch may be envisioned, as discussed in [58]. In such a model system, it was shown that the synchronization due to cell-cell interactions, here at the ectoderm-mesoderm interphase (Fig. 6) may give rise to different signaling rates of the components in the oscillator. This in turn may set up a spontaneous pattern-forming mechanism, which at first is forced to yield the *same* spatial pattern (same segment size) as with the former mechanism, based on the slow-down of the oscillator (Fig. 5). Thus, a simple parameter change in the control system may create such a transition between two, in principle different pattern-forming mechanisms, without inducing lethal effects on the embryo. Once the transition was made, this alternative control system could be exploited, and *Drosophila* may embody this: the Ten-m oscillator, based on binding to cell membranes, would still function in a syncytium with nuclei close to the outer membrane. The transition to a spontaneous pattern-forming mechanism is easily achieved in this system. Indeed, the cooperative binding of Ten-m to the membrane is capable of generating just such a system, as discussed (Fig. 4). This is in accordance with the appearance of the rather ubiquitous expression pattern of the *ten-m* mRNA, but striped localization of the corresponding protein, which indicates that the promoter of *ten-m* is not governed by striped transcription factors. The loss of Ten-m stripes in *ftz* mutants may be due to interactions of Ftz with the kinetics of Ten-m membrane-bound cleavage, through competition with receptor tyrosine kinases. Tyrosine phosphorylation has been found to accompany the cellularization process in space and time (during the formation of the outgrowing membrane) and underlines its importance during the process [79]. The very long nuclear cycle 14 and the gastrulation phase during which stripes form may also reflect a need for the large Ten-m protein to be synthesized. Indeed, *ten-m* can only be transcribed during the long nuclear cycle 14 and later stages [41]. All other nuclear cycles are by far too short, and started transcripts will be aborted [41].

The role of the equidistant Ten-m protein stripes in *Drosophila* is unknown, as is the precise role of *ten-m* in arthropod segmentation. In *Tribolium*, strong knock-down of *Tc-opa* or *Tc-Ten-m* transcripts caused high levels of embryonic lethality, but no overt pair-rule phenotype was observed [80].

The above described transition including a fundamental role for *ten-m* may then have acted as a scaffold on top of which alignment to other pair-rule genes and gap genes may have evolved. Thus, it represents a system which eventually may have developed into the complex hierarchy established for *Drosophila.* A plethora of genes are essential for the final control system. However, it is highly unlikely that this system emerged simultaneously, and so the question is: How did this intricate system evolve from an apparently different kind of system based on sequential segment formation? Thus, it is conceivable that a only minor part of the entire system is the part responsible for this evolutionary transition.

## Conclusions

The *ten-m, opa/zic* control pair may have evolved early. As the membrane-bound Ten-m participates in cell-cell communication, and as its interactions with the *opa/zic* promoter may resemble that of the *Notch* system, it may have been intertwined with the *Notch, wnt* and *FGF* pathways in a putative ancient oscillator-growth mechanism for generation of metameric structures [6]. The dynamics of Ten-m binding to membranes may result in oscillations, and we have shown that the model may be extended to comprise a substantial increase in the period of oscillations, as seen in gene-expression waves recorded for vertebrate segmentation.

While there is much plasticity in the top layers of the hierarchy established for *Drosophila*, if the components are investigated for other insects, the segment- polarity genes *en*, and *wg* are part of a module with much more conserved properties. Equidistant Ten-m protein stripes emerge in *Drosophila* close to this module. It is noteworthy that these stripes are not under transcriptional control, as the mRNA is ubiquitously expressed [41].

The oscillator in Ten-m membrane binding may develop into a syncytial all-or-none spatial stripe generator with minor shifts in the model parameters. The stripe spacing is retained during this transition. The above transition may provide a scaffold, with equally spaced stripes, on top of which the intricate hierarchy with specific stripe cues, established for *Drosophila,* may have evolved. The role of this original scaffold in *Drosophila* segmentation is not clear, as the role of equidistant Ten-m protein stripes in *Drosophila* is unknown, but at least remnants of the scaffold are still present. This indicates that the role of *ten-m* in arthropod segmentation may be substantially more profound than hitherto realized.

### Supplement text

Only few studies exist on the role of *ten-m* in other arthropods. We suggest that *ten-m* and the conserved module of the segment-polarity genes such as *wg* and *en* are better starting points for understanding arthropod segmentation than the top-down approach based on the *Drosophila* hierarchical components. Here, additional arguments from this new viewpoint are presented that may be of interest to experimentalists working on segmentation.

### Gap gene cues

Gap gene interactions and cue establishment have been successfully simulated for quite some time, reviewed in [11, 81], but outside *Drosophila* similar studies are much more recent [82]. Gap genes are expressed early in the control of segmentation of short germ-band insects and control some pair-rule genes, but not necessarily in the domain where the particular gap gene is expressed [4]. A renaming of gap genes to ‘cardinal genes’ has thus been suggested [83]. The interactions of the gap genes in *Drosophila* are quite different to those recorded in the intermediate germband insect (hemipteran) *Oncopeltus fasciatus* [84]. The role of gap gene to mediate cues for defining stripes seem to be an evolutionary late acquisition of dipterans. Such cues are present in *Tribolium*, but they show no relation to *Drosophila* cues [85], and the known number of gap genes does not seem sufficient to specify all the presumptive necessary cues [33]. In a much closer relative to *Drosophila*, the malaria mosquito *Anopheles*, the position of some gap genes are inverted with respect to each other, hence, presumptive cues have undergone substantial evolution even within dipterans [86]. A similar result is found in a study of another basal dipteran, the moth midge *Clogmia albipunctata*, where it appears that stripes of *eve* in the posterior part of the embryo are not under control of the gap-cues recorded in *Drosophila* [82].

### Control of secondary pair-rule genes

In this section, we review a number of reports on the control of secondary pair-rule genes in arthropods. Some reports have doubted the top-down model developed for *Drosophila* with the essential stripe formation mediated from the primary pair-rule genes to the secondary pair-rule genes, reviewed in [10].

*prd* belongs to the PAX group of genes and *pax3/7* plays a role in neurogenesis, segmentation and appendage formation, acquired at the root or prior to the arthropod lineage [38, 87, 88]. Gene expression patterns in spiders are compatible with the presence of gene expression waves, and thus to oscillatory expression. A link between *pax3* and *zic* with *wnt* during neural crest determination has also been demonstrated in vertebrates [89]. *pax* genes may predate the origin of nerve and sensory cells [90, 91].

*sloppy-paired* belongs to the equally ancient fork-head gene family, and *sloppy- paired* is inferred to be crucial in the *en, wg* border module, as studied in *Drosophila* [92, 93].

The intricate relation between secondary pair-rule genes themselves is still a matter of substantial discussion [94]. Indeed, it appears that the regulatory interactions of pair-rule genes are different in the beetle *Tribolium*, with *eve, runt* and *odd* as a three-component module regulating each another, as well as regulating downstream targets such as *prd* and *slp* [95]. Interestingly, *hairy* is not a major player in this regulation. This result was confirmed by [96], where considerable divergence of *hairy* function between *Tribolium* and *Drosophila* was reported.

The role of secondary pair-rule genes in lower arthropods was illuminated by the study of *odd, opa, sip* and *pairberry* in the spider *Cupiennius salei* [97]. Stripes were reported to move from the posterior growth zone toward the anterior, compatible with an oscillator mechanism. Furthermore, *hairy* and *pairberry (Pax III)* started posteriorly and moved all the way to the anterior and *eve* and *runt* were recorded in stripes posteriorly, but were not present anteriorly, while *odd*, *opa* and *sip* were expressed only in the anterior part.

The importance of the regulation of other primary pair-rule genes by *hairy* was questioned [98]. It came as a surprise that Hairy was not found to bind other pair-rule promoters than that of *prd* [98], however, it was suggested that *prd* may be a good candidate for mediating *hairy’s* role on segmentation. *prd* itself had a profound effect on the single promoter element for all-or-none late *eve* stripes [99]. Such a control module may have had an important early role in the evolution of arthropod segmentation.

The elements that generate the expression pattern of a gene were traditionally studied using reporter constructs in transgenic animals. However, this experimental approach has its limits; often, enhancer elements do not faithfully recapitulate native expression patterns. The recorded multiple layers of complexity in *cis*-regulatory regions of developmental genes may indicate, that the usual approach, where regulatory elements are studied in isolation may be up for revision. An early report of disperse versus compact control elements appeared in [100], a comprehensive review is given in [101]. These reports indicate that the original hierarchy paradigm with striped input from *hairy* and other primary pair-rule genes may need to be modified, as studies on the secondary pair-rule genes indicated that these genes regulate the primary pair-rule genes as well [10].

Recently, some progress was also made by using *in silico* models simulating the evolution of patterning during development that can be used to investigate the forces and constraints that helped shape these two developmental modes [102]. Analyses were done on a series of earlier simulation studies, thereby exploiting the similarities and differences in their outcomes in relation to model characteristics. Subsequently, these were used to investigate the circumstances and constraints that were important for the evolution of sequential and simultaneous segmentation modes. The report in [102] suggested that constraints arising from the growth process and spatial patterning signal (in this case posterior elongation producing a propagating wave front versus a tissue-wide morphogen gradient) and the evolutionary history (here referred to as ancestral versus derived segmentation mode) strongly shaped the two segmentation mechanisms.

### The last common ancestor of bilaterians

Originally, Hox and ParaHox genes were believed to be responsible for the emergence of patterning of the A-P axis [103]. However, many *wnt* genes were discovered in sponges, and *wnt* genes (expressed in association with the oral or animal pole) may have provided the initial evolution of A-P axis organization [104, 105]. It was argued that A-P and D-V patterning mechanisms, and a number of gastrulation genes (including *beta-catenin, brachyury, snail, twist, decapentaplegic, bmp* and *fork-head* were in place at least before the common ancestor of cnidarians and bilaterians [104, 105].

Key genes involved in early embryo segmentation are also involved in segmentation of the neuronal network. Organization in segmental neuromeres may have been the main ancestral role. Neurons target other neurons or muscle cells and in the search for such a target, guidance molecules are used. Some typical guidance molecules are cell-adhesion or cell-cell communication molecules similar to those needed for morphogenesis, and they may have been recruited to this at a later time point [106].

Neurons seem to have arisen at the root of sponges and cnidarians [107–109]. Segmentation in the form of axial growth coupled to an oscillator may have arisen as early as these systems. The much studied *Notch* signaling system may have been recruited to this process. The interaction of NICD (the intracellular small part of Notch) with Suppressor of hairless (Su(H)) to derepress *hairy* may be such a common regulatory linkage, preserved from a deep ancestor before cnidarian/bilaterian divergence, perhaps as early as 600–700 Ma ago [110, 111].

As far as the role of the much less studied cell-cell communication system based on *ten-m* is concerned, neuronal protein expression of teneurins (the vertebrate homologs of *ten-m*) was shown to be conserved from vertebrates to flies [112]. This implies a fundamental role during neurogenesis [112] and neuronal pathfinding [43]. The Ten-m interaction with Opa/Zic repression is involved in both *Hydra* and vertebrates. This is a further indication that neural development evolved only once [113].

Finally, in a theoretical study, it was shown that a few cell-cell interacting genes and signaling proteins suffice to create a fair number of spatial patterns, but a small increase results in a complexity threshold generating very many possibilities [114].

### Vertebrate-like models for arthropods

Attempts to search for basic control elements which are less sensitive to cues, but closer to the *en, wg, hh* module and in analogy with the oscillator-growth model, were not successful so far. A simple ancient bistable switch may be embedded in the interactions of the secondary pair-rule genes [14]. Several mutually repressive gene pairs are present in this module (i.e. *eve, odd* and *slp* mutual repression, or that of *odd* and *prd*)*.* The finding of mutual inhibition between *sip* and *en* may be important for its role in such a simple ancient switch [93, 115]. In line with a possible original role of segmentation in a primitive nervous system, it was found that the role of *en* during neurogenesis is very ancient, possibly for the regulation of connectivity of neurons by control of cell adhesion molecules [116]. Thus, the basic mechanism of an oscillator coupled to a bistable switch may be a recurrent theme in arthropod segmentation [25], but redundant systems of cell-cell interactions (i.e. where *ten-m* replaced *Notch*) and varying degrees of overlapping bistable modules may be at play.

### Transition from oscillator-growth to all-or-none stripe formation

In [117], it was argued that anterior spider segments were specified almost simultaneously from a pre-existing field of cells, whereas a vertebrate-like mechanism involving *wnt8* and *Notch/Delta* signaling was used to pattern posterior segments. Also in centipedes, the first five anterior segments were formed almost simultaneously [118], whereas the next 35 plus segments formed sequentially. This supported the hypothesis that short-germ arthropods employ two distinct mechanisms to segment their anterior and posterior body parts. Another hypothesis was based on a study of anterior and posterior segmentation in the intermediate germband insect, *Oncopeltus fasciatus* [119]. Thus, it was suggested that simultaneous specification of all segments as seen in long germband insects such as *Drosophila* might be due to an expansion of the anterior specification mechanism to the posterior part of the embryo.

## Methods

Canton-S embryos were collected, fixed with 4% formaldehyde, dechorionated and devitellinized as described [41]. Embryos were stained with rabbit-anti-Ftz antibodies at 1:300 and mab 113 against Ten-m [41] at 1:250 as described [120]. Secondary antibodies were conjugated to Alexa 488 nm (Ftz) and Alexa 555 nm (Ten-m) fluorochromes, respectively, as described [120].
